# Quail Genomics: a knowledgebase for Northern bobwhite

**DOI:** 10.1186/1471-2105-11-S6-S13

**Published:** 2010-10-07

**Authors:** Arun Rawat, Kurt A Gust, Mohamed O Elasri, Edward J Perkins

**Affiliations:** 1 University of Southern Mississippi, Dept. of Biological Sciences, Hattiesburg, MS, USA; 2 U.S. Army Corps of Engineers, Environmental Laboratory, EP-P, Vicksburg, MS, USA

## Abstract

**Background:**

The Quail Genomics knowledgebase (http://www.quailgenomics.info) has been initiated to share and develop functional genomic data for Northern bobwhite (*Colinus virginianus*). This web-based platform has been designed to allow researchers to perform analysis and curate genomic information for this non-model species that has little supporting information in GenBank.

**Description:**

A multi-tissue, normalized cDNA library generated for Northern bobwhite was sequenced using 454 Life Sciences next generation sequencing. The Quail Genomics knowledgebase represents the 478,142 raw ESTs generated from the sequencing effort in addition to assembled nucleotide and protein sequences including 21,980 unigenes annotated with meta-data. A normalized MySQL relational database was established to provide comprehensive search parameters where meta-data can be retrieved using functional and structural information annotation such as gene name, pathways and protein domain. Additionally, blast hit cutoff levels and microarray expression data are available for batch searches. A Gene Ontology (GO) browser from Amigo is locally hosted providing 8,825 unigenes that are putative orthologs to chicken genes. In an effort to address over abundance of Northern bobwhite unigenes (71,384) caused by non-overlapping contigs and singletons, we have built a pipeline that generates scaffolds/supercontigs by aligning partial sequence fragments against the indexed protein database of chicken to build longer sequences that can be visualized in a web browser.

**Conclusion:**

Our effort provides a central repository for storage and a platform for functional interrogation of the Northern bobwhite sequences providing comprehensive GO annotations, meta-data and a scaffold building pipeline. The Quail Genomics knowledgebase will be integrated with Japanese quail (*Coturnix coturnix*) data in future builds and incorporate a broader platform for these avian species.

## Background

Northern bobwhite represents an avian wildlife species that is critical to the maintenance of ecosystem function. The munitions compound (MC) 2,6-dinitrotoluene (2,6-DNT) contaminates soil and water on military training facilities posing a hazard to Northern bobwhite, a ground foraging bird. Advancing our knowledge in the potential hazards of 2,6-DNT to avian species is important to insure the protection of these wild populations. The utility of Northern bobwhite in various ecological [1], experimental [2] and regulatory [3] scenarios has gained recognition for the species as an excellent experimental avian wildlife model. 

Genomic information for Northern bobwhite lags behind other avian model species with 1717 ESTs derived from a brain-tissue cDNA library [4].  This low coverage of the Northern bobwhite genome limits application of toxicogenomic approaches for assessing the systemic impacts of chemical stressors.  In comparison, avian species such as chicken and zebra finch have been robustly described and microarrays have been developed to allow in-depth investigations [5,6]. For example, various public repositories [7-9] host protein, gene ontology and pathway information for chicken in addition to specialized chicken databases which are also freely accessible [10-12].   

We recognized the need to greatly expand and integrate the information-base for Northern bobwhite to develop the species as a robust avian-wildlife genomic model. The Quail knowledgebase contains 478,142 raw ESTs, assembled nucleotide and protein sequences, and 21,980 unigenes annotated with meta-data. Data entities such as protein information and gene ontology annotation are integrated in a common platform with a web interface for comprehensive parameter searching. The linkage among these data entities is provided by unigene ID that connects the entities internally allowing the user to perform flexible query searches. The result of our effort is a web-accessible knowledgebase for Northern bobwhite which includes user friendly navigation tools and provides EST assembling information, sequence and structural properties and complex search utilities, bundled with an alternative method to generate sequence scaffolds to “stitch” transcripts against a reference organism. The data represented in the Quail Genomics knowledgebase have provided novel insights into the systemic perturbations of 2,6-DNT in Northern bobwhite [13]. Similarly, the knowledgebase can provide researchers the ability to perform analysis and curate genomic information to further their own research pursuits.

## Construction and content

### *Platform architecture*

**Figure 1:**
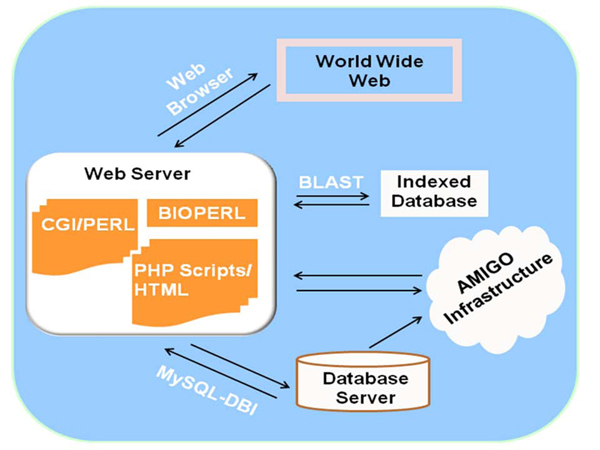
**The overview of Quail Genomics web architecture.** Various in-house Perl, CGI, Bioperl and HTML scripts are stored in webserver and retrieve information from database to visualize results in web browser. The Amigo infrastructure is locally installed and interacts with local database and webserver.

The Quail Genomics knowledgebase is a web-based tool implemented with PERL 5.10.0, CGI, PHP 5.3, and BioPERL 1.6 script programs developed in-house interfacing with MySQL 5.4.3 database [14] through PERL-DBI and integrate with class packages and modules of Go-Dev project [8] (Figure 1). The user interface is supported in HTML that is hosted on Apache 2.2.13 webserver [15] (UNIX version). The Quail Genomics knowledgebase currently runs on a duo 2.26GHz Quad core Intel Xeon (Intel Corporation, Santa Clara, CA) that uses the 64 bit Snow Leopard v1.6 (Apple Computer Inc. Cupertino, CA) operating system. The assembled sequences and chicken protein sequences are indexed and the annotation information is stored in the database. Users can access various features and data by PHP, PERL/CGI-BIN scripts hosted in Apache and retrieve information from database and/or indexed text files to display results. Hyperlinks are provided on the display results for retrieval of additional information.

### *Database schema and implementation*

**Figure 2:**
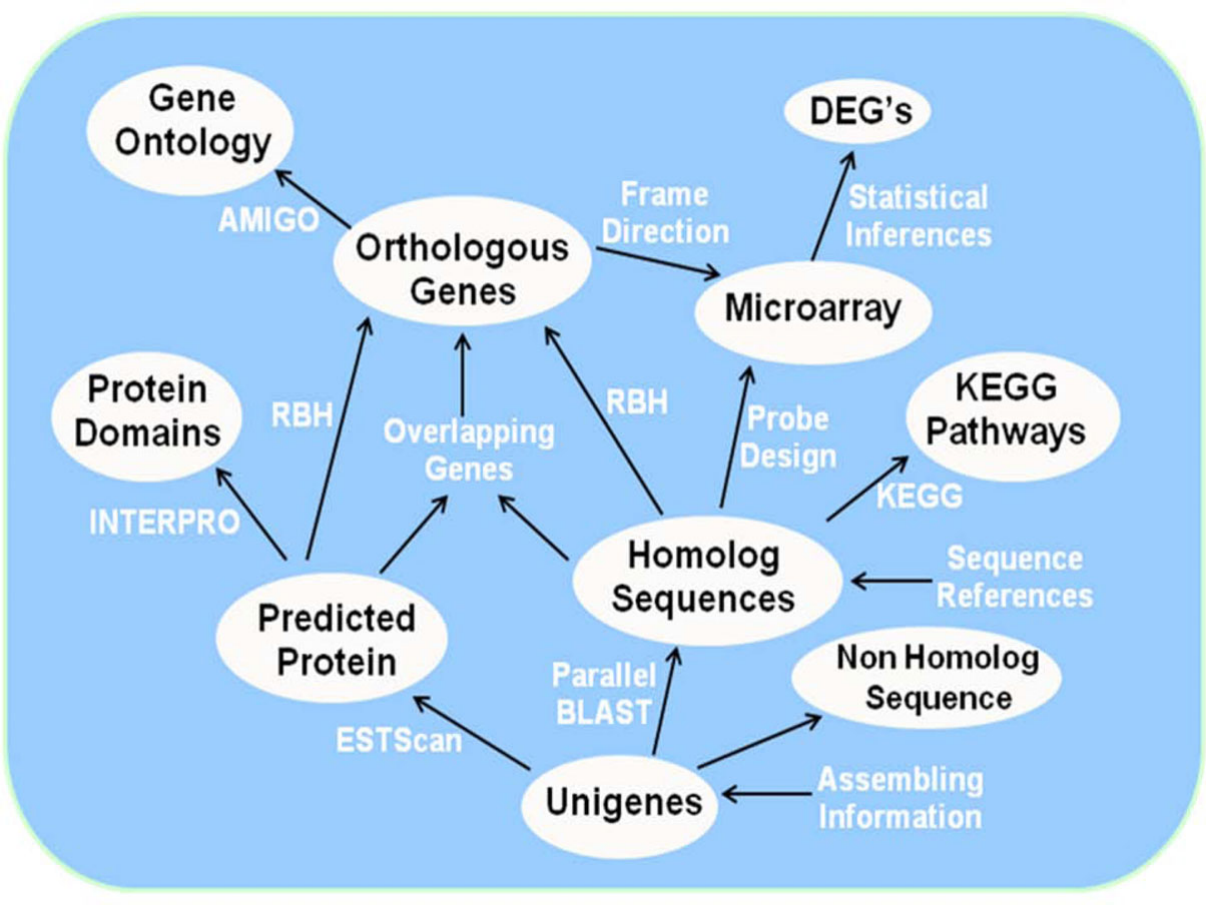
**The data-flow diagram and interaction among the data entities stored in database.** DEG, differentially expressed gene; RBH, reciprocal BLAST hit.

The conceptual data representation of annotation and association of data entities is summarized in Figure 2. The database schema design and development was performed based on this interaction among the data entities and is stored in the relational database. The protein database represents Refseq sequences for chicken downloaded from Entrez in fasta format (http://www.ncbi.nlm.nih.gov/sites/gquery).  The assembled sequences and corresponding chicken protein sequences are indexed for fast querying and stored as flat files.  

### *Nucleotide assembly and annotation*

The preparation of nucleotide assembly data resulted in 71,384 unique sequences [13]. All sequences were annotated against non-redundant protein database with BLASTX and also against model organism and chicken database using Parallel Blast [16] with HPC[17].  Prediction of protein-coding regions was established using ESTScan which uses a hidden Markov model to identify coding regions that uses *Gallus gallus* as training data [18].  The predicted protein-coding regions were scanned for protein signatures using Interproscan for high throughput annotation [19,20]. The output is stored in the database and can be retrieved via querying for visual inspection of protein domains in HTML format. Hyperlinks are provided on the display results for retrieval of additional information. Microarray-based gene expression data is also included in the Quail Genomics knowledgebase where *p*-value and fold changes are stored as persistent data for each experiment.  A full description of microarray experiments and analyses is provided in Rawat et al [13].

### *Gene Ontology browser*

We performed reciprocal blast hit (RBH) to assess ortholog detection among Northern bobwhite and chicken (*Gallus gallus*, an intra-order phylogenetic relative of Northern bobwhite) across both the putative homologs (BLASTX, E ≤ 10^-5^) and predicted ORFs (ESTScan) that might lead to orthologous genes (Figure 2). We observed that putative homolog-ORF pairs (where matching protein identifiers were sorted on minimum E value for blast hits) were complementary in 85% of comparisons to the RBH pairs. To maximize the ortholog count for the Northern bobwhite, we conjoined ortholog sets derived from each method resulting in non-redundant orthologous unique transcripts that represent 8,825 unique gene products (Figure 2). To functionally annotate the 8,825 Northern bobwhite orthologs, we investigated and inferred putative GO [21] annotations finding matching annotations for 4,786 (54.2%) genes. We implemented the GO-Dev project from Amigo locally to provide Tree Browser to represent the Northern bobwhite orthologs. 

GO-Dev is an open-source platform that consists of CGI/perl modules, database structure and web interface [22]. GO-Dev and dependencies including GraphViz [23] which are required to successfully run the Amigo infrastructure locally were downloaded and installed. The Amigo infrastructure runs on the Quail Genomics webserver and interacts with our local MySQL database where database dumps are imported into the GO table structures.  We exported our GO data for Northern bobwhite orthologs to the local Amigo database and the data can be viewed via tree browser from Quail Genomics.

### *Genomic scaffolds*

The 71,384 unigenes identified for Northern bobwhite are an over representation of the total protein-coding genes that are expected to make up the Northern bobwhite genome. Frequently, due to missing EST sequences, the ESTs from a single gene may not overlap to assemble to a contiguous sequence resulting in non-overlapping contigs and singletons or splits in genes. Also, stringent parameters during assembly of ESTs into contigs might lead to unassembled sequences especially when the sequences have low genome coverage [24]. These issues of missing sequences or fragmentation might lead to partial representation of a protein-coding sequence. Many of the sequence fragments might actually represent the same protein leading to redundancy in the assembled sequences. 

For model organisms, methods such as Blat [25] and Bowtie [26] can be utilized to annotate against a reference genome; however inadequate representation of a reference sequence for a non-model organism makes annotation difficult. Other methods like Ensemble gene-build pipeline [24] are also available however these do not allow user to select ‘gene of interest’ and allow visualization. Finally, methods such as Genescript [27] do provide visualization features, however integration of these with our web interface and database is not practical due to workflow and operating system incompatibility. 

**Figure 3:**
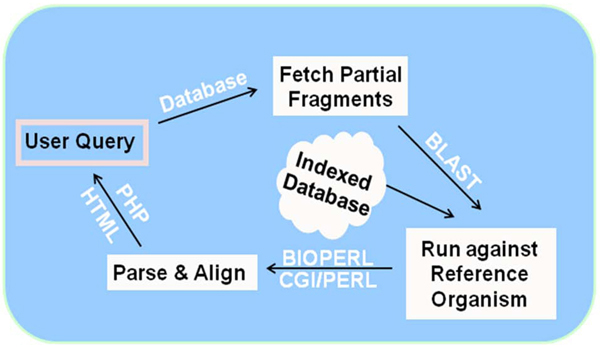
**The flow chart representing the building of genomic scaffolds.** The user selects gene of interest and the query retrieves the Northern bobwhite unigenes stored in the database, maps to the reference organism and visualize in web browser.

Therefore, we have built a pipeline that generates scaffolds by aligning unigenes that might represent partial sequence fragments against specific coding regions of gene to generate scaffolds consisting of multiple-unigenes. The user can select from the list of all the Northern bobwhite genes stored in the database that have more than six unigenes/fragments (arbitrarily set) that represent same protein-coding region. The scaffolds can be built by clicking ‘gene of interest’ which fetches these similar unigene fragments from the database.  These are aligned against the protein database of chicken and visualized in a web browser (Figure 3). As described above, the protein database consists of Refseq sequences for chicken and stored in our local server. The output of BLAST includes information including:  start position, end position, frame direction and sequence homology against the reference sequence for each unigene which is parsed and extracted with BioPerl script. This information is used to align each unigene that might represent a partial fragment against the chicken protein sequence.  

It may be of interest to further study these scaffolds to understand redundancy or potential alternate spliced elements. In conjunction with Parallel Blast output stored as persistent data and the sequence information stored in relational database management system (RDBMS), our pipeline allows users to interact and visualize results ‘on the fly’. The temporary file handler added in the script allows multi-user access and deletes files created during scaffold building, to maintain housekeeping on the server. 

## Utility and discussion

Quail Genomics knowledgebase hosts the genomic data for Northern bobwhite which includes nucleotide and protein sequences, meta-data properties and microarray expression data as summarized in Table 1. The knowledgebase provides a central repository for storage, data management and access through a web interface. 

**Table 1:** Composition of data available in Quail Genomics knowledgebase.

*Data Content in Quail Genomics*	
**Sequencing**	478,142 EST
**Post assembly**	71,384 Unigenes (35,904 contigs, 35,480 singlets)
**Putative homologs**	21,912 hits
**Predicted protein regions**	39,400 potential ORFs
**Protein domains**	15,057 Interproscan hits
**Ortholog detection**	8,825 putative orthologs
**Gene Ontology**	4,786 GO terms

### *Annotation search*

**Figure 4:**
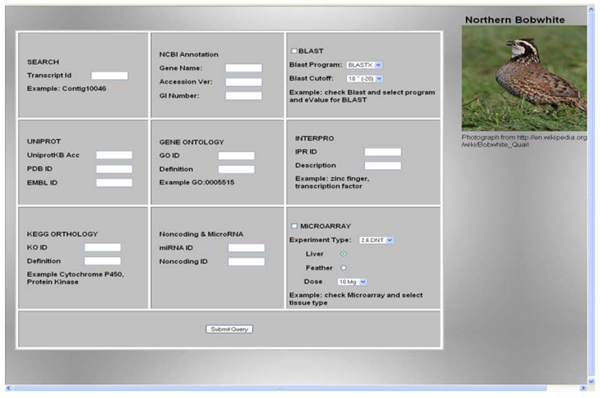
Web browser results of query search options for the Quail Genomics.

**Figure 5:**
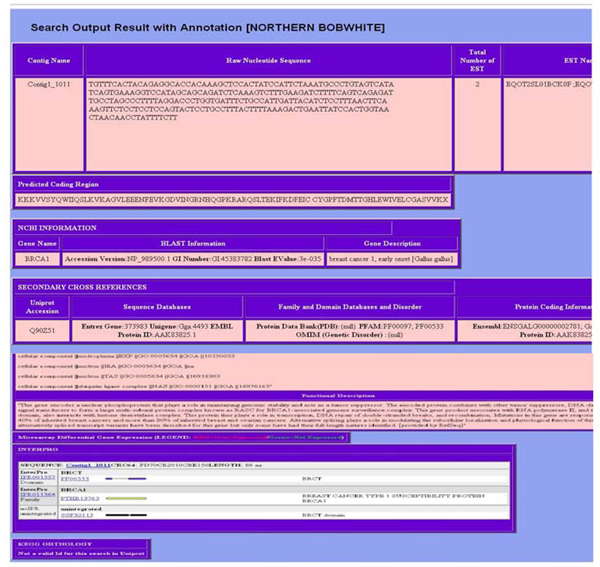
Results of the output in the browser executed after performing a parameter search.

Users can access sequence information (Figure 4) through single query searches (i.e. unigene ID) and batch search (i.e. by blast hit cutoff or microarray experiment). We cross referenced the various entities of data with internal ID that allow comprehensive annotation search with gene name, protein and regular expression search (i.e. cytochrome p450) that might be of interest to specific users. While browsing through any search (i.e. differentially expressed gene for an experiment), the user can click hyperlinked unigene ID to see the detailed report. The output is provided in tabular form with assembling, sequence, structural properties and metadata (Figure 5).   

### *Additional searches*

Beside annotation search, we have integrated additional searches in our platform. Users can search ESTs that assemble as contigs and visualize the overlap and direction against the assembly. Users can also input their sequence and perform blast search (BLASTN, TBLASTX) against the indexed nucleotide sequences of Northern bobwhite. The expression data is stored based on experiment and dose and output can be viewed for microarray probes sorted on *p*-value. The microarray probes which had a statistically significant increase or decrease in expression (*p*-value<.05) are highlighted in red or green, respectively. 

### *GO browser*

The Northern bobwhite orthologs are functionally annotated under the Inferred from the electronic annotation (IEA) evidence level. The GO categories of these orthologs can be browsed for biological processes, cellular components, and molecular functions through the GO Tree Browser implemented from Amigo [28]. With guidelines as defined by GO consortium [29], these orthologs are candidates that might be considered for update to Inferred from Sequence Orthology (ISO) evidence level.

### *Genomic scaffolds*

**Figure 6:**
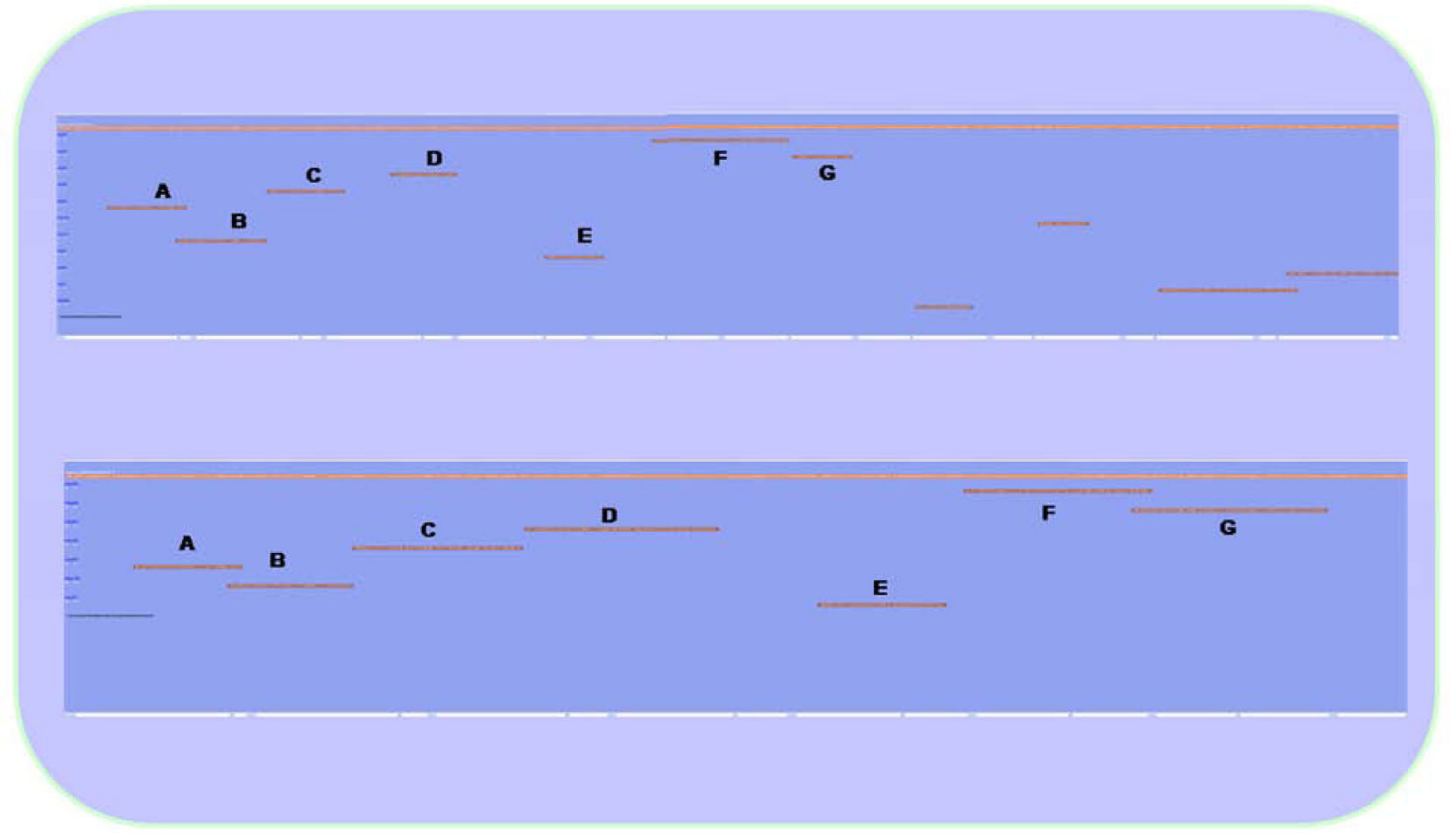
**The figure depicts the non overlapping sequences lead to split among the unigenes representing same gene.** The upper panel shows Northern *bobwhite* unigenes alignment against the chicken protein sequence for gene F5. The lower panel is alignment against same gene with the Northern *bobwhite* predicted protein. The labels in both panels represent same unigenes for raw nucleotide and predicted protein and roughly expanse the same region of protein-coding chicken sequence. * The magnification of the two panels is different

The user can select parameter BLASTP for predicted proteins from ESTScan and BLASTX for nucleotide sequences and *e-value* cutoff to visualize scaffolds for the Northern bobwhite unigenes.  All the unigenes that comprise more than six fragments are listed in the web page with annotation. Clicking on the gene of interest will show frame direction along with sequence alignment against the indexed *Gallusgallus* Refseq protein sequence data (downloaded February 2008). Northern bobwhite unigenes alignment against the chicken protein sequence for gene F5 is shown for raw nucleotide and predicted protein (Figure 6). This example explains the redundancy in the assembled sequences, stemming from the fragmentation of unigenes due to non-overlapping contigs and singletons. The unigenes representing the same protein-coding sequence of chicken fragmented due to either absence of overlapping sequences or insufficient assembling parameter threshold. We also believe that this information can also be utilized not only to quality control sequencing coverage for an individual gene coding region but also to study alternate splicing mechanism. Also, more meaningful full-length protein-coding sequence can be built either by tuning *e-value* cutoff or cross matching alignment of raw nucleotide with the predicted protein output. One other advantage of using the scaffold visualized in web browser is that it allows multiple users to access a central system without separate installation of dependencies and software(s) and local databases.

### *Past applications and results*

The data represented in Quail Genomics knowledgebase have provided insights into the metabolic perturbations underlying several observed toxicological phenotypes in a 2,6-DNT-exposure case study investigating Northern bobwhite [13]. The comprehensive metadata attributes helped us to identify RT-qPCR validated impacts.

### *Future developments*

Japanese quail, a reproductive avian model species [30], has only 136 ESTs available in GenBank (May’2010). The Quail Genomics knowledgebase will soon incorporate 559,819 ESTs generated for Japanese quail by next generation sequencing using GS-FLX technology (454 Life Sciences / Roche, Branford, CT) developed to further ecotoxicological research in Japanese quail (unpublished). Subsequent updates will be performed every 3 months or as required. We will continue to work on the scaffold module allowing users to interact with output and download in GFF format.

## Conclusions

The Quail Genomics knowledgebase provides a web-based utility for genomic investigations in an emerging wildlife model, the Northern bobwhite. As of March 2010, this knowledgebase contains raw sequence, assembled sequence, annotations and gene expression data for the Northern bobwhite. The Quail Genomics knowledgebase will be integrated with Japanese quail genomics and incorporated into a broader platform for investigations of avian species.

## Availability and requirements

Quail Genomics knowledgebase is publicly available [31].  The web interface is HTML 4.01 and has been tested with Firefox 3 and Internet Explorer 7.  PERL, PHP, GO-DEV, MYSQL, BioPERL 1.6 are supported by dependencies and run on Quad core, 16GB RAM, MAC OSX 1.6. Raw data and microarray data have been deposited in public repositories and can be downloaded from the links provided.

## Competing interests

The authors declare that there are no competing interests.

## Authors' contributions

AR designed and developed the database. AR performed programming in PERL, PHP, HTML, BIOPERL and integration of GO-DEV libraries and web interface development, Webserver administration and drafted the manuscript.

KAG developed the normalized cDNA library for Northern bobwhite, facilitated the sequencing effort, was involved in coordination of bioinformatics effort and assisted in manuscript development. 

MOE involved in coordination of bioinformatics effort. 

EJP participated in its design, coordination and manuscript writing. 

All authors read and approved the final manuscript.
